# Analysis of different outcome parameters and quality of life after different techniques of free vascularized lymph node transfer

**DOI:** 10.1016/j.jvsv.2024.101934

**Published:** 2024-06-24

**Authors:** Lisanne Grünherz, Carlotta Barbon, Donata von Reibnitz, Epameinondas Gousopoulos, Semra Uyulmaz, Pietro Giovanoli, Diana Vetter, Christian Alexander Gutschow, Nicole Lindenblatt

**Affiliations:** a Department of Plastic Surgery and Hand Surgery, University Hospital Zurich, Zurich, Switzerland; b Department of Visceral and Transplant Surgery, University Hospital Zurich, Zurich, Switzerland

**Keywords:** Omentum flap, Vascularized lymph node transfer, Quality of life, PROM, VLNT

## Abstract

**Objective:**

Vascularized lymph node transfer (VLNT) has become an important surgical technique in the treatment of lymphedema. Considering the different available regions available for flap harvest, we aimed to analyze different donor sites for VLNT with respect to donor site morbidity, impact on limb volume, and patient-reported outcome measurements (PROMs).

**Methods:**

A single-center prospective study of all patients undergoing VLNT at the Department of Plastic Surgery and Hand Surgery of the University Hospital Zurich between September 2016 and 2023 was conducted. Lymph nodes were harvested either from the omentum (gastroepiploic [GE]-VLNT), the lateral thoracic wall (LTW), or the superficial inguinal region (SI-VLNT). Volume measurements and PROMs were assessed preoperatively and at different postoperative intervals.

**Results:**

Overall, 70 patients with upper limb lymphedema (21%) or lower limb lymphedema (79%) with different lymphedema stages were included. There were 49 patients who underwent GE-VLNT, followed by LTW-VLNT (n = 16) and SI-VLNT (n = 5). Lymph node harvest from the SI was associated with a significantly higher frequency of seroma development. The average percentage volume loss related in comparison to the preoperative volume of the affected limb was 9% after GE-VLNT, 10% after LTW-VLNT, and 5% after SI-VLNT without a significant difference between the groups. PROMs revealed significant improvements for physical functioning, symptoms and psychological well-being, with no differences between the VLNT techniques.

**Conclusions:**

VLNT leads to a significant improvement of quality of life and can decrease limb volume effectively, regardless of the selection of donor site. GE-VLNT has become our flap of choice owing to its low donor site morbidity and its properties that allow a double transplantation while avoiding a second donor site.

**Article Highlights**

• **Type of Research:** Single-center prospective nonrandomized cohort study
• **Key Findings:** Overall, 70 patients with lymphedema received a free vascularized lymph node transfer, harvested from different donor sites. Although lymph node harvest from the superficial inguinal region was associated with a significantly higher frequency of seroma development, there were no differences regarding postoperative volume loss and improvements of quality of life between the different donor sites.
• **Take Home Message:** A free vascularized lymph node transfer leads to a significant improvement of quality of life and can decrease limb volume effectively in patients with lymphedema, regardless of the selection of donor site for flap harvest.

Reconstructive lymphatic microsurgery has become one of the most important milestones in lymphedema treatment, with a growing body of literature demonstrating a significant and lasting reduction of limb volume.^1^, ^2^, ^3^, ^4^, ^5^, ^6^, ^7^ Nowadays, lymphovenous anastomoses (LVAs) as well as free vascularized lymph node transfer (VLNT) have become a standard in the multimodal therapy of patients with lymphedema in most countries. Most surgeons prefer a VLNT either in patients with advanced lymphedema or in patients with primary lymphedema in whom LVAs have the risk to damage functioning lymph vessels or simply be unsuccessful, owing to the underlying lymphatic phenotype.^8^ Regarding postoperative outcomes, a previous meta-analysis has shown significantly better long-term outcomes after VLNT in comparison with LVAs in terms of the greater likelihood of discontinuing compression garments.^9^

In principle, a VLNT can be prepared from any major lymph node site, sparing the lymph nodes essential for drainage of that limb by performing reverse lymphatic mapping. Available donor sites include supraclavicular lymph nodes, submental nodes, superficial inguinal nodes, lateral thoracic nodes, mesenteric lymph nodes, and gastroepiploic (GE) lymph nodes.^8^ Although the exact mechanism by which VLNT improves lymphedema is remains unclear, two theories have been advanced. First, the secretion of lymphangiogenic cytokines from the VLNT transplant leads to formation of spontaneous efferent and afferent lymphatic connections (lymphangiogenesis) between the transferred lymph node package and the recipient site, resulting in a partial restoration of lymph drainage. According to the second theory, the lymph node flap acts as a sponge that absorbs the lymph into the lymph nodes and redirects it into the venous system through the formation of lymphovenous channels.^8^

To date, the choice of donor site seems to be based primarily on the surgeon's preference in addition to patient-specific factors. In fact, despite numerous studies on outcomes after VLNT, there is only limited evidence on whether a specific donor site is superior in terms of morbidity and postoperative outcomes. Based on our prospective registry including all patients who underwent lymphatic reconstructive surgery at our department, the aim of this study was to analyze different donor sites for VLNT with respect to its donor site morbidity, impact on limb volume and patient-reported outcome measurements (PROMs).

## Methods

We conducted a prospective, nonrandomized monocenter study including all patients undergoing lymphatic reconstructive surgery at the Department of Plastic Surgery and Hand Surgery of the University Hospital Zurich between September 2016 and 2023. Approval was given by the Cantonal Ethics Committee of Zurich, Switzerland (Ethical approval Nr.: 2020-0011 and 2018-00,284). Written consent was obtained from all the patients or their parents in the case of minors. Patients who were unable to fill in questionnaires owing to insufficient knowledge of the German language, impaired psychointellectual abilities, or any psychiatric disorder were only included for baseline and treatment variables. All patients had to adhere to a formal and strict conservative therapy protocol for ≥1 year. This protocol includes the wear of fitted compression garments and an optimal support by physiotherapists, including regular complete decongestive therapy and an intensive treatment phase in the month before the surgery.

### Volume measurements and calculations

All patients received manual circumference measurements by the physiotherapist with subsequent calculation of limb volume according to the method described by Kuhnke,^10^ preoperatively and at standardized intervals (2 weeks, 6 weeks, 6 months, and 12 months) after the surgery, according to our standard regime. If patients were operated at two different anatomical locations, such as both legs, each leg was considered independently to allow a more precise assessment of the outcome.

The percentage of volume loss was calculated using the following formula:

$$\frac{\left(\text{Preoperative}\;\text{volume}\;\text{affected}\;\text{limb}\;–\;\text{Postoperative}\;\text{volume}\;\text{affected}\;\text{limb}\right)}{\text{Preoperative}\;\text{volume}\;\text{affected}\;\text{limb}}$$

Owing to the relatively high number of patients with bilateral lymphedema, calculation of excess volume loss, which is based on the volume of the healthy contralateral limb, was not possible. Patients for whom the preoperative volume was either missing or was identified as unreliable (eg, because it was taken too many months before the surgery), were not taken into consideration for calculation of volume changes.

### PROMs

To evaluate the patient-reported outcome, patients with upper limb lymphedema (ULL) completed the LYMPH Q Upper Extremity Module and Short Form Health Survey 36 (SF 36) preoperatively and at the aforementioned intervals. Patients with lower limb lymphedema (LLL) received the Lymphoedema Functioning, Disability and Health Questionnaire for Lower Limb Lymphedema (LYMPH-ICF-LL) and SF-36 at the same intervals. Use of the LYMPH Q Upper Extremity Module, authored by Drs Klassen, Pusic, and Cano, was made under license from Memorial Sloan Kettering Cancer Center (New York, NY). Additionally, we collected patient characteristics, demographic data, and surgical details.

### Statistical analysis

Data were then analyzed using Microsoft Excel Version 14.3.6 (Microsoft Corp., Redmond, WA). and GraphPad Prism Version 7.04 (GraphPad, La Jolla, CA). A *t* test or one-way analysis of variance was performed to compare for continuous parametric data. Fisher's exact test was performed to compare two categorial variables and χ^2^ test for three or more categorial variables. A *P* value of less than .05 was defined as significant.

## Results

**Fig 1:**
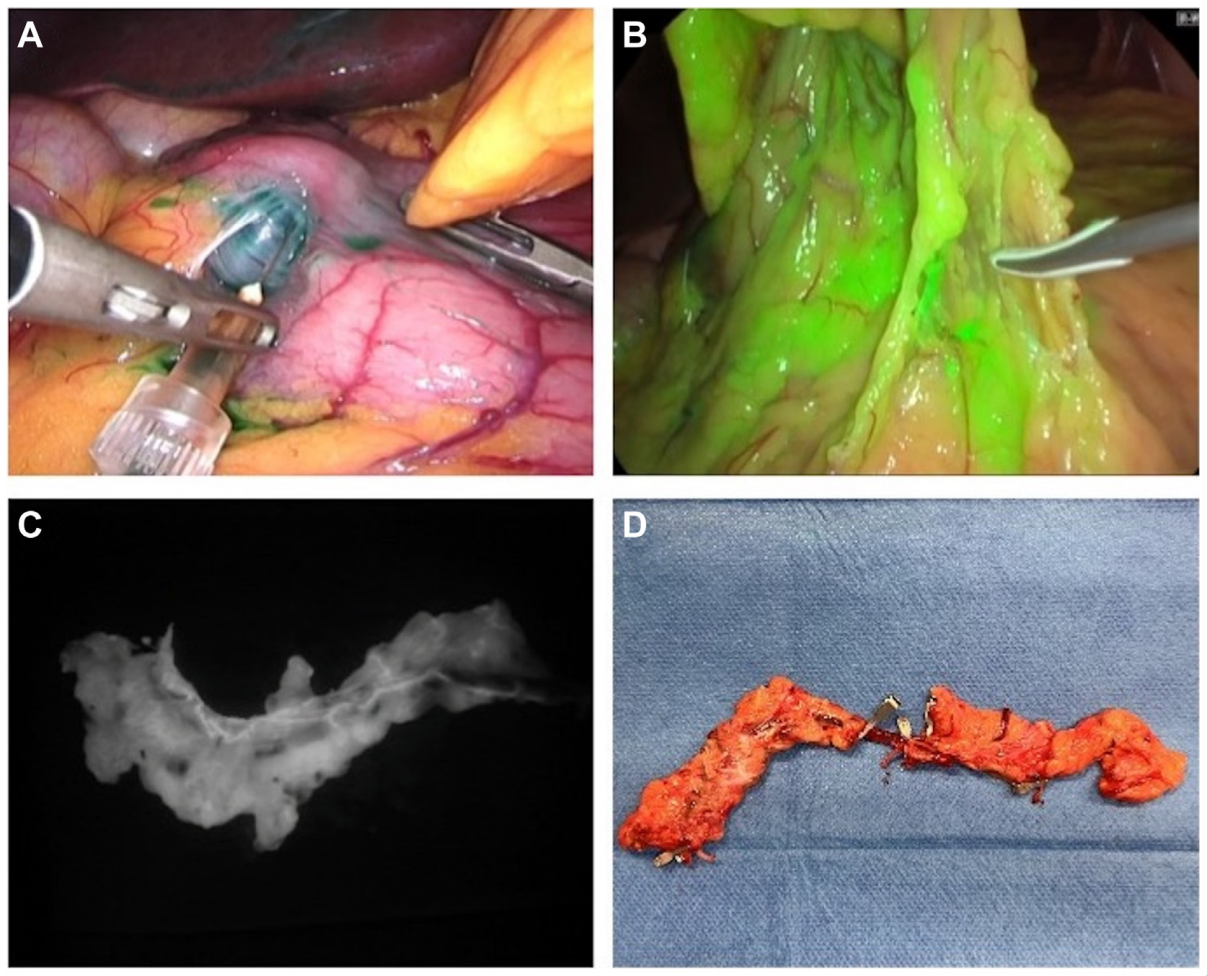
**(A)** Gastroepiploic (GE) vascularized lymph node transfer (VLNT) harvest. Intraoperative injection of indocyanine green into the gastric wall to mark **(B)** lymph nodes and lymphatics of the omentum. **(C)** Near-infrared image of the GE lymph node flap ex vivo, showing lymph nodes and lymph vessels along the GE vessels. **(D)** Preparation of the GE vessles before dividing the flap to enable double VLNT.

**Fig 2:**
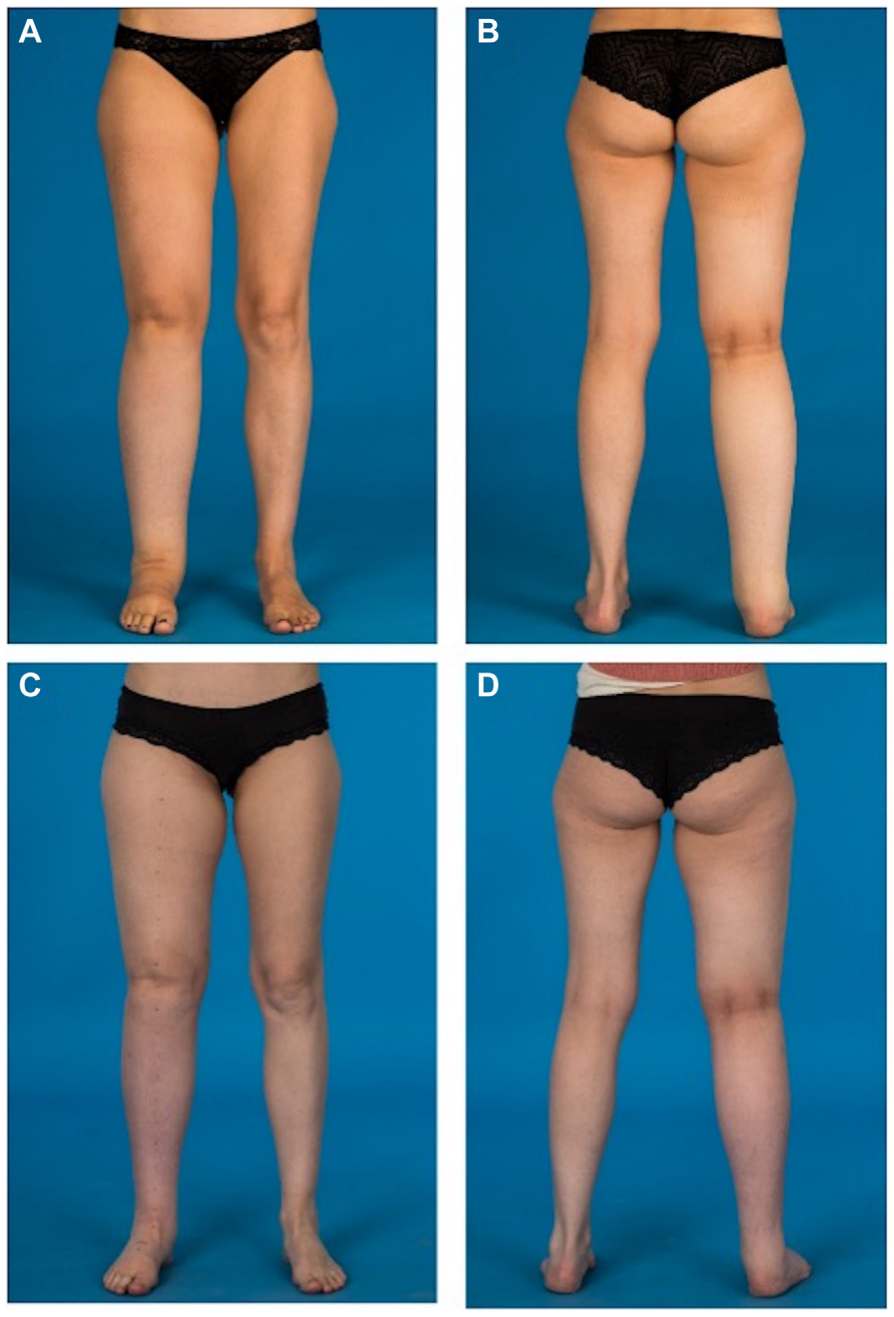
**(A** and **B)** Preoperative photograph of a 39-year-old woman with secondary lymphedema of her right leg. The patient received gastroepiploic (GE) vascularized lymph node transfer (VLNT) to the right groin, two lymphovenous anastomoses (LVAs) at the foot and liposuction of the leg (400 mL lipoaspirate). **(C** and **D)** At the 8-month follow-up a significant volume reduction of the right leg was observed.

**Fig 3:**
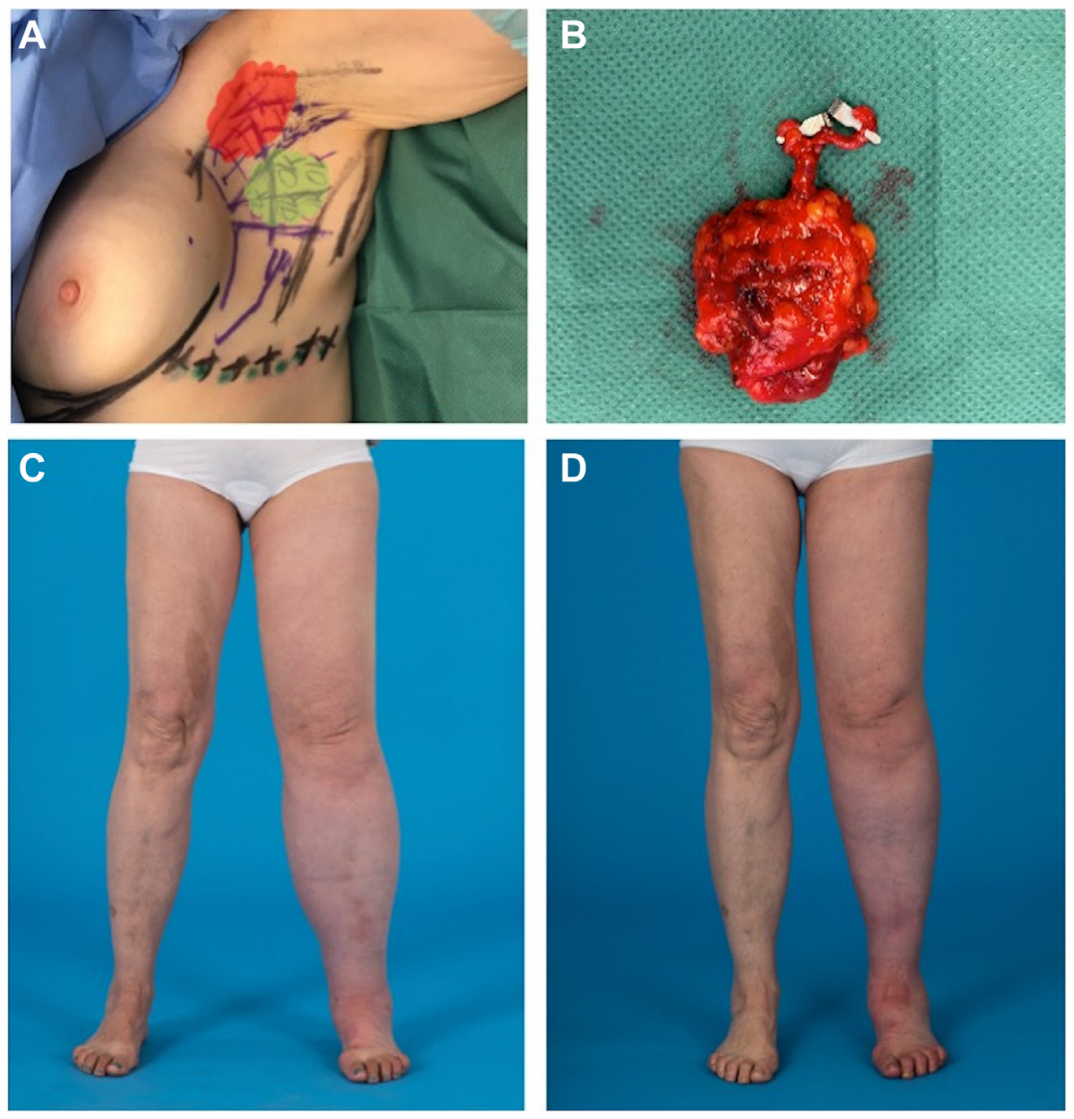
**(A)** Reverse lymphatic mapping for lateral-thoracic wall vascularized lymph node transfer (VLNT). Patients receive preoperative lymph-scintigraphy to mark the lymph nodes draining the arm with technetium (Tc99). Additionally, indocyanine green (ICG) is injected at the lateral thoracic wall (LTW). Then, ICG-positive lymph nodes that drain the LTW (green area) are included in the **(B)** lymph node flap, while Tc99 positive lymph nodes (red area) are spared to preserve lymphatic drainage of the arm. **(C)** Preoperative photograph of a 58-year-old woman with secondary lymphedema of her left leg after pelvic lymph node dissection owing to endometrial cancer. **(D)** The 1-year-follow-up after LTW-VLNT to the left groin, two lymphovenous anastomoses (LVAs) at the foot and liposuction of the thigh (300 mL lipoaspirate) showed a significant volume reduction.

We included 70 patients who underwent GE-VLNT (Fig 1, *A-D*), which was harvested laparoscopically in 49 patients. Fig 2 shows an example of a patient who underwent GE-VLNT. In 21 patients, the lymph node flap was either harvested from the lateral thoracic wall (LTW-VLNT) or the superficial inguinal region (SI-VLNT) after reverse lymphatic mapping (Fig 3, *A-D*). Most patient characteristics showed an even distribution between the groups, except for age and primary lymphedema, whose proportion was significantly higher among patients underwgoing GE-VLNT (Table I).

**Table I:** Patient characteristics

	GE-VLNT	LTW-VLNT	SI-VLNT	*P* value
Patients	49	16	5	NA
Extremities operated	61 (74)	16 (20)	5 (6)	NA
Age, years	41 ± 17	55 ± 10	58 ± 11	.003
BMI, kg/m^2^	26 ± 5	27 ± 5	26 ± 5	.9
Lymphedema stage (operated extremity)^a^				.08
I	14 (23)	1 (6)	0 (0)	
II	42 (69)	10 (63)	4 (80)	
III	5 (8)	5 (31)	1 (20)	
Duration until surgery, years	11.71 ± 10.79	11 ± 9	6 ± 4	.49
Cause of lymphedema				.008
Primary	28 (57)	4 (25)	0 (0)	
Secondary	21 (43)	12 (75)	5 (100)	
Affected anatomical region				NA
Lower extremity	37 (76)	16 (100)	0 (0)	
Upper extremity	10 (20)	0 (0)	5 (100)	
Lower extremity and genitals	2 (4)	0 (0)	0 (0)	
Laterality of disease				.33
Unilateral	34 (69)	12 (75)	5 (100)	
Bilateral	15 (31)	4 (25)	0 (0)	
Recurrent erysipelas	18 (30)	5 (31)	1 (20)	.88
Concomitant disease^a^				
None	29 (59)	12 (75)	3 (60)	.52
Vascular, hypertension, diabetes, coronary artery disease	9 (18)	2 (13)	0 (0)	NA
Venous disease (venous insufficiency, thrombosis, varicose veins)	3 (6)	0 (0)	0 (0)	NA
Metabolic (adipositas, thyroid, metabolic syndrome)	8 (16)	2 (13)	2 (40)	NA
Inflammatory disease	1 (2)	0 (0)	0 (0)	NA
Arthritis/osteoporosis	2 (4)	0 (0)	0 (0)	NA
Pulmonary, asthma	4 (8)	0 (0)	0 (0)	NA
Other syndromes	2 (4)	0 (0)	0 (0)	NA

*BMI*, Body mass index; *GE*, gastroepiploic; *LTW*, lateral thoracic wall; *NA*, not avaiable; *SI*, superficial inguinal region; *VLNT*, vascularized lymph node transfer.

Values are mean ± standard deviation or number (%).

a More than one value is possible for every patient or extremity operated.

The details in regard to the surgical procedures are presented in Table II. Most of the patients (n = 46) undrwent VLNT in combination with LVAs (Table II). Of note, GE-VLNT allowed for simultaneous bilateral VLNT in patients with bilateral lymphedema (24%) or for unilateral double VLNT (29%) to reconstruct lymph drainage at two different anatomical locations, for example, by transplanting one-half of the flap in the inguinal area and the other one-half of the flap in the knee or ankle of the same limb in patients with unilateral lymphedema. In contrast, when harvesting lymph nodes from the LTW or SI, splitting is not possible owing to a considerably smaller flap size. Overall, 30 patients received additional liposuction. There were no statistically significant differences regarding liposuction volume (GE-VLNT, 861 ± 969 mL; LTW-VLNT, 836 ± 204 mL; SI-VLNT, 1300 ± 0 mL; *P* = .68) and hospital duration between the different groups (GE-VLNT, 4 ± 1 days; LTW-VLNT, 4 ± 1 days; SI-VLNT, 4 ± 0 days; *P* = .11). Regarding duration of the surgery, we noted that GE-VLNT took significantly longer (GE-VLNT, 393 ± 69 minutes; LTW-VLNT, 285 ± 40 minutes; SI-VLNT, 312 ± 3 minutes; *P* = .0001).

**Table II:** Operation details

	GE-VLNT	LTW-VLNT	SI-VLNT	*P* value
Surgical technique (operated extremities)				.21
VLNT	30 (49)	4 (25)	2 (40)	
VLNT + LVA	31 (51)	12 (75)	3 (60)	
Double VLNT in patients				.0001
Unilateral	14 (29)	0 (0)	0 (0)	
Bilateral	12 (24)	0 (0)	0 (0)	
Additional liposuction	24 (39)	5 (31)	1 (20)	.26
Liposuction volume per extremity, mL	861 ± 969	836 ± 204	1300 ± 0	.68
Length of surgery, minutes				.0001
VLNT	393 ± 69	285 ± 40	312 ± 3	
VLNT + LVA	416 ± 87	308 ± 69	375 ± 49	
Length of hospital stay, days				.11
VLNT	4 ± 1	4 ± 1	4 ± 0	
VLNT + LVA	5 ± 1	4 ± 1	5 ± 1	
Decrease in compression class (n = 39)^a^	8 (47)	1 (8)	1 (33)	.56

*GE*, Gastroepiploic; *LTW*, lateral thoracic wall; *SI*, superficial inguinal region; *VLNT*, vascularized lymph node transfer.

Values are mean ± standard deviation or number (%).

a Information was available for 39 operated extremities, 17 receiving gastroepiploic VLNT and 22 receiving thoracic/abdominal wall VLNT.

**Fig 4:**
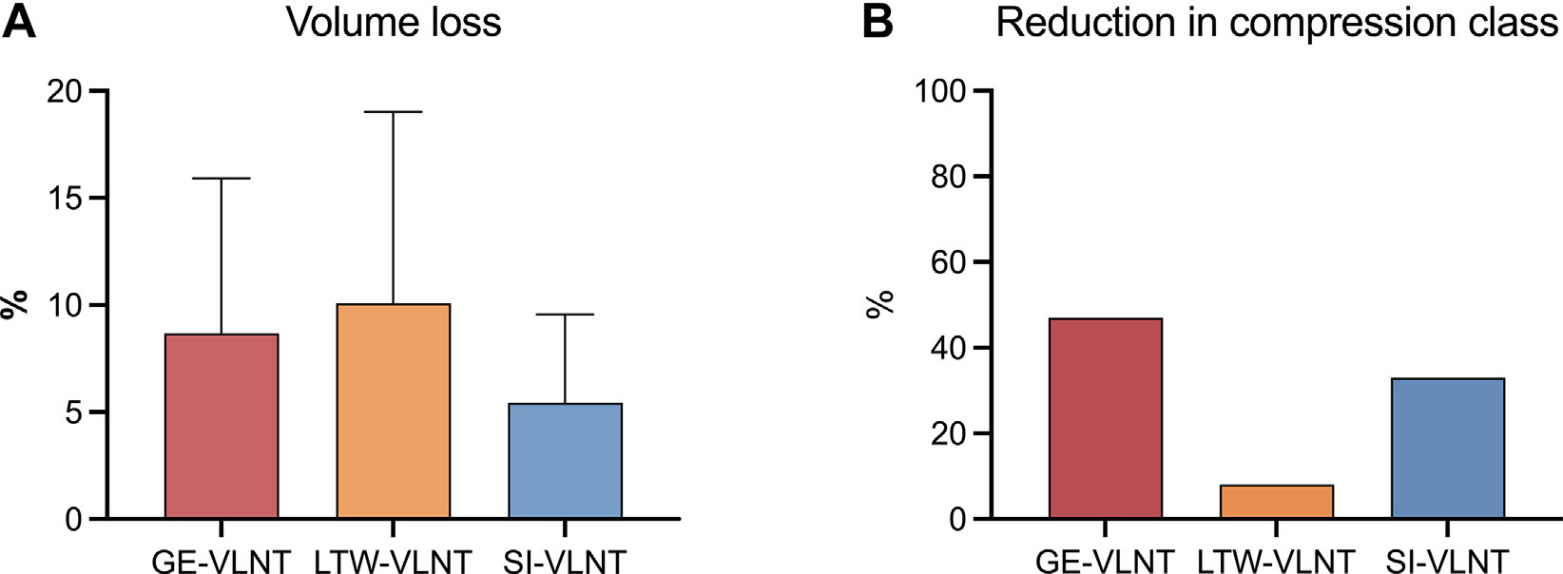
**(A)** Mean volume loss in patients after gastroepiploic (*GE*)-vascularized lymph node transfer (*VLNT*) (8% ± 7%), lateral thoracic wall (*LTW*)-VLNT (10% ± 9%), superficial inguinal region (*SI*)-VLNT (5% ± 4%) without a significant difference between the surgical technique (*P* = .6). **(B)** There was a decrease in compression class in 47% of the patients after GE-VLNT, 8% after LTW-VLNT, and 33% after SI-VLNT, without being statistically significant (*P* = .6).

Overall, 45 extremities (55%) could be evaluated with regard to their volume measurements at the 6- to 12-month follow-up. At this follow-up, 100% of patients with ULL and 63% of patients with LLL for whom the measurements were available showed a volume decrease. In these patients, the average percentage of volume loss was 9% after GE-VLNT, 10% after LTW-VLNT, and 5% after SI-VLNT. Regardless of flap choice, there was no significant difference in percentage of volume loss in patients with LLL compared with ULL (8% ± 7% vs 10% ± 8%). Statistical analysis revealed no significant differences in percentage volume loss between patients after GE-VLNT, LTW-VLNT, or SI-VLNT (Fig 4, *A*). Data on preoperative and postoperative compression class was available for 39 patients, revealing a decrease in compression class in 47% of patients after GE-VLNT, 8% after LTW-VLNT, and 33% after SI-VLNT (Fig 4, *B*). Two patients—one after GE-VLNT and one after SI-VLNT—were able to discontinue wearing compression garments. In patients with a history of recurrent erysipelas, no further episodes were observed after VLNT.

Regarding donor site complications (Table III), lymph node harvest from the SI was associated with a significantly greater frequency of seroma development. Three of five patients treated with harvest from the SI required sclerotherapy. Although not seroma, wound infection, or hematoma were observed after GE-VLNT, one patient suffered a perforated gastric ulcer that was diagnosed 1 month after the laparoscopic flap harvest. After LTW-VLNT harvest guided by reverse mapping, one patient with primary lymphedema developed a mild donor site lymphedema of the arm (grade I) after additional vaccination against the coronavirus disease 2019 (COVID-19), requiring compression therapy.

**Table III:** Donor site complications

Complication (operated patients)	GE-VLNT	LTW-VLNT	SI-VLNT	*P* value
Wound infection/healing issues	0 (0)	1 (6)	0 (0)	.19
Seroma	0 (0)	1 (6)	4 (80)	.001
Hematoma	0 (0)	1 (6)	0 (0)	.18
Other complications	1 (2)	1 (6)	0 (0)	.63

*GE*, Gastroepiploic; *LTW*, lateral thoracic wall; *SI*, superficial inguinal region; *VLNT*, vascularized lymph node transfer.

Values are number (%).

**Fig 5:**
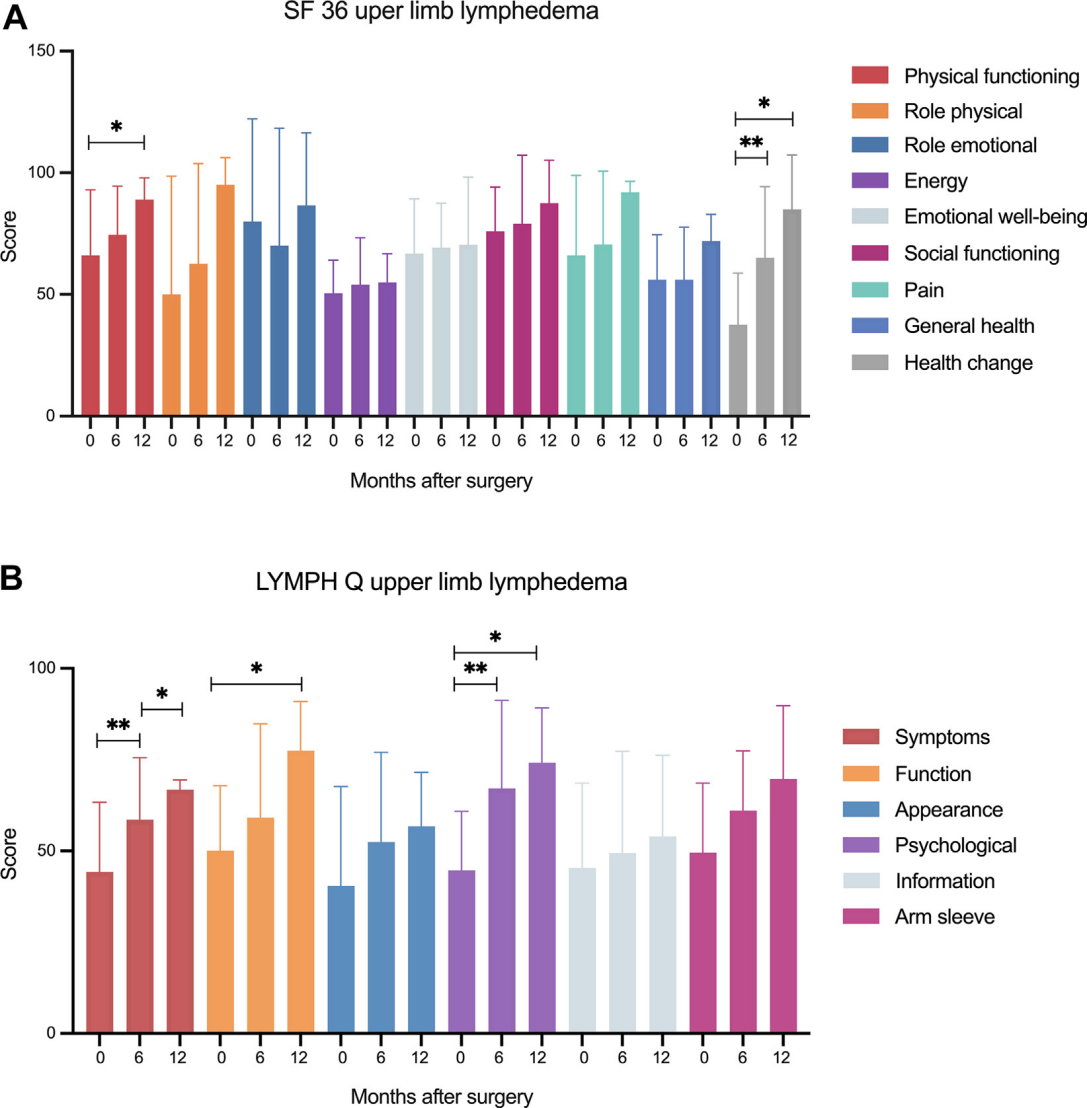
**(A)** Analysis of Short Form Health Survey 36 (*SF-36*) of patients with upper limb lymphedema (ULL) revealed improvements in a broad range of complaints, reaching significance for physical functioning (66.0 ± 26.9 vs 89.0 ± 8.9; *P* = .02) and health change (37.5 ± 21.3 vs 85 ± 22.4; *P* = .01) 12 months postoperatively. **(B)** The LYMPH Q showed significant improvements of symptoms (44.3 ± 19.1 vs 66.8 ± 2.7; *P* = .02), function (50.1 ± 17.8 vs 77.4 ± 13.6; *P* = .04), and psychological well-being (44.7 ± 16.2 vs 74.2 ± 15.0; *P* = .04) at 12 months postoperatively.

A subset total of 47 patients received preoperative and postoperative questionnaires. For patients with ULL, 10 of 11 patients that who reached their 6- or 12-month follow-ups completed their questionnaires. The SF-36 (Fig 5, *A*) revealed improvements in all domains with statistically significant differences in physical functioning (66.0 ± 26.9 vs 89.0 ± 8.9; *P* = .02) and health change (37.5 ± 21.3 vs 85 ± 22.4; *P* = .01). Based on the LYMPH Q (Fig 5, *B*), patients with ULL experienced statistically significant improvements of symptoms (44.3 ± 19.1 vs 66.8 ± 2.7; *P* = .02), function (50.1 ± 17.8 vs 77.4 ± 13.6; *P* = .04), and psychological (44.7 ± 16.2 vs 74.2 ± 15.0; *P* = .04) at 12 months postoperatively. Comparison of SF-36 and LYMPH Q between patients after GE-VLNT and LTW VLNT revealed no significant differences (Supplementary Table I (online only), Supplementary Table II (online only), Supplementary Table III (online only), Supplementary Table IV (online only), online only).

**Fig 6:**
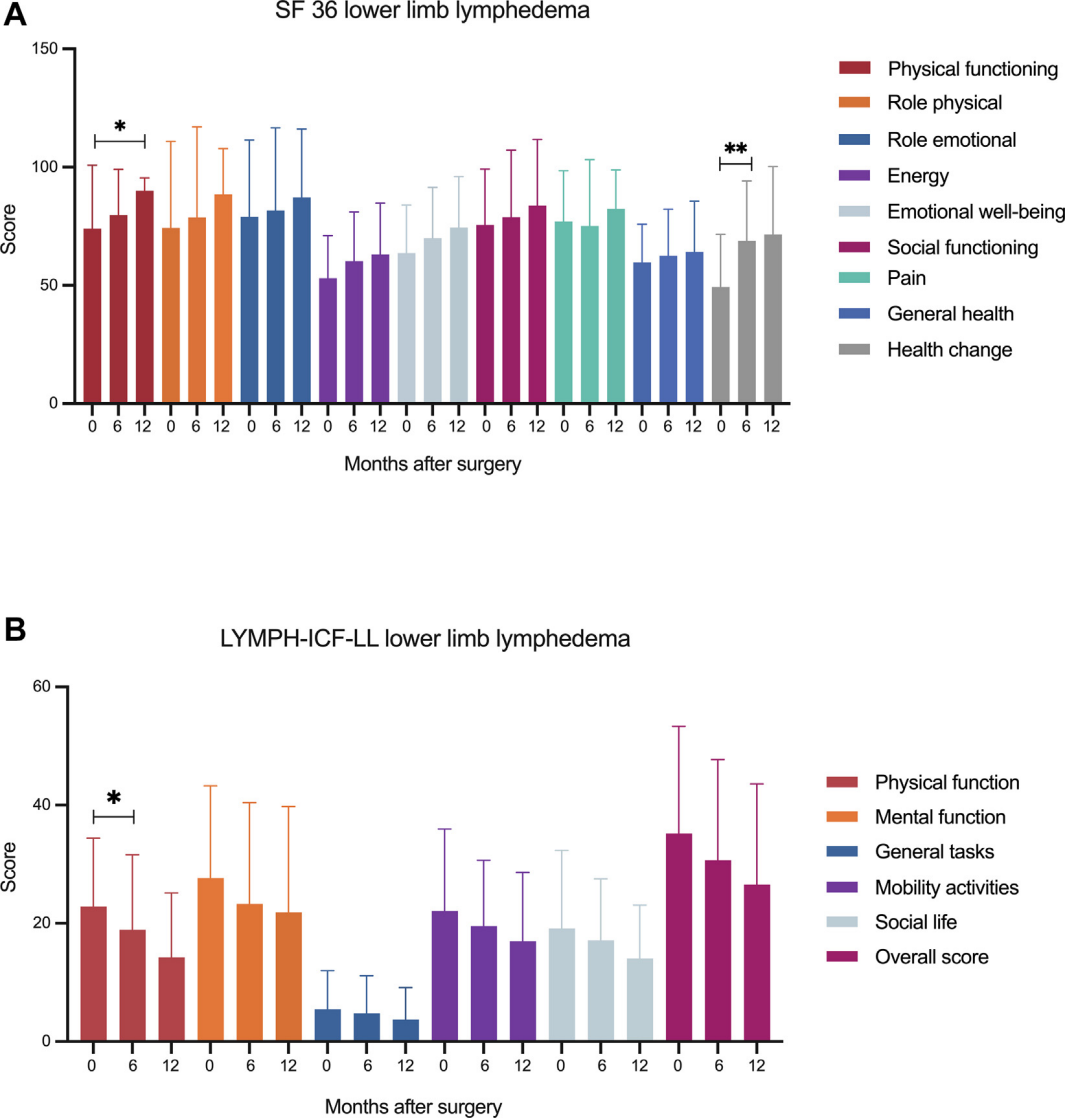
**(A)** Analysis of the Short Form Health Survey 36 (*SF-36*) of patients with lower limb lymphedema (*LLL*) revealed significant improvements for physical functioning (74.0 ± 26.9 vs 90.0 ± 5.4; *P* = .04) and health change (49.3 ± 22.3 vs 71.5 ± 28.7; *P* = .007). **(B)** Results of the Lymphoedema Functioning, Disability and Health Questionnaire for Lower Limb Lymphedema (LYMPH-ICF-LL) demonstrated improvements in all domains of the questionnaire, reaching significance for the physical function of the leg (22.9 ± 11.6 vs 19.5 ± 11.4; *P* = .05).

For patients with LLL, 21 of 36 patients who completed their questionnaires after 6 to 12 months. SF-36 (Fig 6, *A*) showed improvements in all domains 12 months postoperatively with statistically significant differences in physical functioning (74.0 ± 26.9 vs 90.0 ± 5.4; *P* = .04) and health change (49.3 ± 22.3 vs 71.5 ± 28.7; *P* = .007). Similar results were found on the LYMPH-ICF-LL (Fig 6, *B*), with improvements regarding physical function, mental function, general tasks, mobility activities, social life, and overall score. Statistical analysis of SF-36 and LYMPH-ICF-LL in patients after GE-VLNT compared with superficial inguinal VLNT showed no significant differences (Supplementary Table I (online only), Supplementary Table II (online only), Supplementary Table III (online only), Supplementary Table IV (online only), online only).

## Discussion

The results of this study demonstrate that VLNT in patients with lymphedema can decrease limb volume and compression class effectively and leads to a significant improvement of quality of life, independent of the VLNT donor site.

Regarding complications, we observed a significantly greater number of patients with donor site seroma after SI-VLNT. Although none of the patients who developed seroma at the donor site required revision surgery, repeated punctures or even sclerotherapy can be burdensome for patients. Our incidence of donor site seroma is similar to other studies, confirming a higher risk after flap harvest from the superficial inguinal area, particularly when combined with the deep inferior epigastric perforator flap harvest, in contrast with flap harvest from the LTW.^11^^,^^12^

One patient with late-onset primary lymphedema of the lower extremities was diagnosed with a mild donor site lymphedema of the arm 6 months after the VLNT was harvested from the thoracic wall. Importantly, the occurrence of arm lymphedema was chronologically closely related to COVID-19 Spikevax vaccination. Considering the current literature, the risk for donor site lymphedema remains low, but still ranges from 1.5% to 12.5%, with a remarkably higher incidence after LTW VLNT.^13^^,^^14^ However, there is growing evidence that lymph node flap harvest can lead to a transient impairment of lymphatic drainage of the limb.^15^^,^^16^ Based on postoperative lymphoscintigraphy, Viitanen et al^15^ observed a minor impairment in lymphatic function without clinically evident lymphedema in 60% of the patients after superficial inguinal VLNT. Even after axillary sentinel lymph node biopsy, a transient lymphatic dysfunction can occur in a subset of patients.^17^ Although the COVID-19 Spikevax vaccination per se may provoke transient lymphedema,^18^, ^19^, ^20^ it may have acted as a second trigger in addition to a potentially compromised lymphatic function after flap harvest from the thoracic wall.

In contrast, GE lymph node harvest does not bear the risk of donor site lymphedema owing to the abundancy of lymphatic tissue in the omentum. Unfortunately, one of our first patients was diagnosed with a perforated gastric ulcer 1 month after flap harvest, necessitating laparoscopic closure of the perforation. This result prompted us to introduce standardized postoperative prophylaxis using a proton pump inhibitor for 3 months, after which no further patient developed stomach perforation or any gastric symptoms that were deemed surgery related. Considering the risk of donor site lymphedema and the incidence of postoperative seroma, particularly after SI-VLNT, we changed our practice and almost exclusively perform GE-VLNT since 2021. Furthermore, the size of the GE flap allows either a double unilateral or bilateral transplantation, avoiding a second donor site. Although this concept is relatively new, there is growing evidence that a double inset and the corresponding segmental treatment of the affected extremity can influence volume reduction positively, as well as different PROMs such as the LYMQoL questionnaire.^21^^,^^22^ The potential disproportionate volume of the transplanted inset in comparison with recipient location has prompted the use of split-thickness skin grafting at the distal recipient site,^23^ such as the medial malleolus. In our view, split-thickness skin grafting can be avoided by carefully trimming the flap, upon and based on visualization of the perfusion by indocyanine green. This approach is indispensable to prevent compromised flap perfusion or even wound dehiscence caused by the excessive flap volume.

Our results are in line with the existing literature, demonstrating no significant difference among the different flaps in terms of their efficiency to induce volume reduction and prevent infectious episodes.^24^^,^^25^ In contrast, a previous meta-analysis found that “extra-abdominal donor sites” decreased limb volume more effectively than “intra-abdominal donor sites.” However, because the authors themselves considered most of the studies to be of poor methodological quality, their results should be interpreted with caution.^26^ Among the limitations of this study is the relatively small size of the SI-VLNT flap group and the increased presence of bilateral lymphedema, which prevents us from calculating the excess volume loss or circumference decrease rate, as commonly seen in the literature.

The results of this study once again emphasize that lymphatic reconstructive microsurgery succeeds in offerring an improvement in quality of life with respect to a broad range of physical and psychological complaints, reaching significance for the improvement of physical functioning and lymphedema-related symptoms. These results further confirm our previous study, demonstrating the impact of lymphatic reconstructive microsurgery on patient quality of life by achieving an improvement of lymphedema-related complaints in all patients, independent of the extent of volume loss of the operated extremity.^27^ Additionally, this study indicates comparable clinical outcomes independent of the anatomical location of the donor flap. Similar results have been presented in a recent meta-analysis comparing different outcomes after extra-abdominal vs abdominal VLNT. The authors of the meta-analysis concluded that there is no difference regarding improvements of quality of life with respect to the different donor sites, a finding in line with our experience.^26^ A similar observation was made by Schaverien et al, who reported on equally improved patient-reported outcomes after mesenteric, groin, lateral thoracic, GE-VLNT, and submental VLNT, adding to an increasing body of evidence with comparable findings.^25^

## Conclusions

The results of this study emphasize that a VLNT leads to a significant improvement in quality of life and can decrease limb volume effectively, as well as the compression class in patients with lymphedema, regardless of the selection of donor site for VLNT. Considering the analysis of donor site complications, GE lymph node transfer has become our flap of choice. Owing to its low donor site morbidity and its abundancy of lymphatic tissue, it enables a segmental treatment approach of the extremity or bilateral transplantation through a double VLNT, sparring the disadvantages of a second donor site.

## Author Contributions

Conception and design: LG, CB, DR, EG, SU, PG, DV, CG, NL

Analysis and interpretation: LG, CB, DR, EG, SU, PG, DV, CG, NL

Data collection: LG, CB, DR

Writing the article: LG, CB

Critical revision of the article: DR, EG, SU, PG, DV, CG, NL

Final approval of the article: LG, CB, DR, EG, SU, PG, DV, CG, NL

Statistical analysis: LG, CB

Obtained funding: Not applicable

Overall responsibility: NL

LG and CB contributed equally to this article and share co-first authorship.

## Figures and Tables

**Supplementary Table I (online only):** Comparison of Short Form Health Survey 36 (*SF-36*) domains in patients with lower extremity lymphedema 12 months after gastroepiploic (*GE*) vascularized lymph node transfer (*VLNT*) versus lateral thoracic wall (*LTW*)-VLNT

SF-36 domain	GE-VLNT	LTW-VLNT	*P* value
Physical functioning	82.8 ± 20.2	91.3 ± 8.6	.6
Role physical	82.8 ± 35.0	81.3 ± 23.9	.4
Role emotional	81.2 ± 36.5	91.8 ± 16.6	.5
Energy	57.8 ± 26.2	66.3 ± 11.1	.1
Emotional well-being	70.3 ± 23.5	72.0 ± 25.9	.8
Social functioning	81.3 ± 29.2	69.0 ± 38.7	.8
Pain	76.2 ± 27.0	80.7 ± 13.6	.3
General health	61.3 ± 17.6	67.5 ± 29.0	.4
Health change	72.2 ± 24.0	56.3 ± 37.5	.8

Values are mean ± standard deviation.

**Supplementary Table II (online only):** Comparison of LYMPH ICF LL domains in patients with lower extremity lymphedema 12 months after gastroepiploic (*GE*) vascularized lymph node transfer (*VLNT*) versus lateral thoracic wall (*LTW*)-VLNT

LYMPH ICF LL domain	GE-VLNT	LTW-VLNT	*P* value
Physical function	17.5 ± 12.9	11.5 ± 9.8	.8
Mental function	25.1 ± 17.9	19.5 ± 20.3	.3
General tasks	3.8 ± 6.6	6.0 ± 6.0	.4
Mobility activities	20.3 ± 13.9	15.0 ± 16.0	.8
Social life	15.3 ± 11.3	19.5 ± 5.4	.4
Overall score	30.8 ± 21.9	26.3 ± 13.6	.7

Values are mean ± standard deviation.

**Supplementary Table III (online only):** Comparison of Short Form Health Survey 36 (*SF-36*) domains in patients with upper extremity lymphedema 12 months after gastroepiploic (*GE*) vascularized lymph node transfer (*VLNT*) versus superficial inguinal region (*SI*)-VLNT

SF-36 domain	GE-VLNT	SI-VLNT	*P* value
Physical functioning	76.3 ± 23.1	82.5 ± 10.6	.5
Role physical	56.3 ± 43.8	100.0 ± 0.0	.4
Role emotional	66.7 ± 47.2	100.0 ± 0.0	.4
Energy	51.3 ± 20.1	50.0 ± 21.2	.6
Emotional well-being	51.3 ± 20.1	58.0 ± 48.1	.5
Social functioning	76.9 ± 31.0	81.3 ± 26.5	.5
Pain	68.4 ± 33.3	90.0 ± 0.0	.5
General health	57.5 ± 24.5	65.0 ± 0.0	.5
Health change	62.5 ± 32.7	100.0 ± 0.0	.5

Values are mean ± standard deviation.

**Supplementary Table IV (online only):** Comparison of LYMPH Q domains in patients with upper extremity lymphedema 12 months after gastroepiploic (*GE*) vascularized lymph node transfer (*VLNT*) versus superficial inguinal region (*SI*)-VLNT

LYMPH Q domain	GE-VLNT	SI-VLNT	*P* value
Symptoms	56.1 ± 16.1	68.0 ± 4.2	.3
Function	60.5 ± 29.1	74.0 ± 8.5	.6
Appearance	52.3 ± 21.3	63.0 ± 25.5	.6
Psychological	60.4 ± 21.3	81.5 ± 26.2	.3
Information	57.2 ± 22.8	55.5 ± 33.2	.9
Arm sleeve	55.7 ± 11.2	88.0 ± 17.0	.1

Values are mean ± standard deviation.

## Appendix

Additional material for this article may be found online at www.jvsvenous.org.
